# Interpretable deep learning survival predictions in sporadic Creutzfeldt–Jakob disease

**DOI:** 10.1007/s00415-024-12815-1

**Published:** 2024-12-16

**Authors:** Johnny Tam, John Centola, Hatice Kurucu, Neil Watson, Janet MacKenzie, Alison Green, David Summers, Marcelo Barria, Sohan Seth, Colin Smith, Suvankar Pal

**Affiliations:** 1 The UK National CJD Research and Surveillance Unit, Centre for Clinical Brain Sciences, Chancellor’s Building, University of Edinburgh, Edinburgh, EH16 4TG UK; 2 Institute of Adaptive and Neural Computation, School of Informatics, University of Edinburgh, Edinburgh, EH8 9AB UK

**Keywords:** Creutzfeldt–Jakob disease, Sporadic, Prion, Survival, Neural network, Deep learning, Artificial intelligence

## Abstract

**Background:**

Sporadic Creutzfeldt–Jakob disease (sCJD) is a rapidly progressive and fatal prion disease with significant public health implications. Survival is heterogenous, posing challenges for prognostication and care planning. We developed a survival model using diagnostic data from comprehensive UK sCJD surveillance.

**Methods:**

Using national CJD surveillance data from the United Kingdom (UK), we included 655 cases of probable or definite sCJD according to 2017 international consensus diagnostic criteria between 01/2017 and 01/2022. Data included symptoms at diagnosis, CSF RT-QuIC and 14-3-3, MRI and EEG findings, as well as sex, age, *PRNP* codon 129 polymorphism, CSF total protein and S100b. An artificial neural network based multitask logistic regression was used for survival analysis. Model-agnostic interpretation methods was used to assess the contribution of individual features on model outcome.

**Results:**

Our algorithm had a c-index of 0.732, IBS of 0.079, and AUC at 5 and 10 months of 0.866 and 0.872, respectively. This modestly improved on Cox proportional hazard model (c-index 0.730, IBS 0.083, AUC 0.852 and 0863) but was not statistically significant. Both models identified codon 129 polymorphism and CSF 14-3-3 to be significant predictive features.

**Conclusions:**

sCJD survival can be predicted using routinely collected clinical data at diagnosis. Our analysis pipeline has similar levels of performance to classical methods and provide clinically meaningful interpretation which help deepen clinical understanding of the condition. Further development and clinical validation will facilitate improvements in prognostication, care planning, and stratification to clinical trials.

## Introduction

### Background

Sporadic Creutzfeldt–Jakob disease (sCJD) is the commonest human prion disease, with a worldwide annual incidence of 1–2 per million [1, 2]. Other aetiologies of human prion disease include inherited prion disease (IPD), iatrogenic CJD (iCJD), and variant CJD (vCJD) associated with dietary exposure to bovine spongiform encephalopathy (BSE) in cattle. Due to their status as transmissible spongiform encephalopathies (TSE), human prion diseases including sCJD are the focus of international public health concern and disease surveillance [2].

sCJD is fatal and characterised by rapid disease progression, with median disease duration between 4 and 5 months [1]. Key clinical features at presentation include rapidly progressive dementia, visual disturbance, neuropsychiatric disturbance and motor decline. Diagnosis can be established using the 2017 International CJD Surveillance Network diagnostic criteria [3]. The adoption of this criteria, alongside advances in the diffusion-weighted magnetic resonance imaging (MRI) [4] and real-time quaking-induced conversion assay (RT-QuIC) [5], has been shown to have significantly improved the diagnosis of sCJD [6].

### Prognostication in sCJD

There is a significant degree of clinical heterogeneity in sCJD. Although most people diagnosed with sCJD are deceased 5 months from symptom onset, a significant portion have slower disease progression and survive beyond 12 months [7]. Symptoms are also variable, with distinct clinical presentations reported including rapidly progressive dementia, neuropsychiatric onset, stroke-like presentations, and the Heidenhain subtype characterised by prominent visual disturbance [2, 8].

Prognostication in sCJD therefore remains a significant challenge. Effective predictions of progression and survival will offer helpful information to healthcare professionals, families, carers and patients on the management of the illness and care planning. This is especially important given the introduction of evidence-based palliative care pathways in prion disease [9]. Finally, as novel compounds enter investigations in prion disease clinical trials, effective survival prediction will facilitate stratification for trial inclusion and interpretation of survival benefit after interventions [10].

### Pre-mortem determinants of survival in sCJD

A polymorphism at codon 129 of the prion protein (*PRNP*) gene is a major determinant of susceptibility to sCJD, and heterogeneity in clinical and neuropathological phenotypes [7, 11, 12]. Valine homozygosity (VV) and methionine/valine heterozygosity appear to confer longer disease duration compared to the methionine homozygosity (MM), the commonest codon 129 polymorphism. Age of onset has also been reported as an important determinant of survival, with varying levels of influence [13–17]. The hypothesis that younger age confers a greater survival benefit is intuitive, given that younger individuals may have better physiological reserves. The presence of periodic sharp wave complexes (PWCs) on electroencephalogram (EEG) and CSF 14-3-3 have also been identified as possible predictive markers of survival [17]. Prion protein (PrP^Sc^) isotype was also shown to influence survival, but this variable is only available via neuropathologic examination conducted post-mortem.

Overall, these determinants of survival provide a challenging picture for clinicians to interpret. Moreover, improvements in the diagnosis of sCJD have increased case ascertainment [6, 18], identifying cases that were previously missed or misdiagnosed. An updated, comprehensive, and clinically relevant survival prediction model is needed.

Several published studies have reported survival or prognostic prediction models in sCJD [12, 19, 20]. We will discuss these models alongside our own new model.

### Survival analysis methods

#### Cox proportional hazard model

The Cox proportional hazard model (CoxPH) is a widely used multivariate survival analysis method in clinical and biomedical research [21]. In a recent benchmark of survival analysis methods in ‘-omics’ and clinical data, it performs remarkably well against other statistical methods and recent machine-learning techniques [22]. However, two important limitations may reduce the effectiveness of CoxPH, particularly where the focus is survival prediction. CoxPH relies on the proportional hazard assumption due to its reliance of an arbitrary baseline hazard function. This assumption is commonly violated, especially in clinical data [23]. Violation of the proportional hazards assumption can reduce the power of the model, especially when survival prediction is the primary task. Despite this, the presence non-proportional hazards are rarely reported in clinical studies of survival analysis [24]. Furthermore, the CoxPH model is a linear model, which assumes that the relationship between features and the log of the relative hazards is linear. Therefore, the CoxPH model is unable to capture more complex interactions amongst predictors.

#### Artificial neural networks (ANN)

New methods have been proposed as alternatives to the CoxPH model, either by modelling survival differently or by extending the CoxPH model. With advances in computational power and data science, deep-learning methods such as artificial neural networks (ANNs) offer new and performant methods of analysing and predicting survival [22, 25]. Within survival analysis, several novel methods have been proposed using ANNs, such as DeepSurv which combines an ANN with the CoxPH model [26]. As the volume and complexity of health data continue to increase, survival analysis using ANNs has great potential to facilitate novel and performant predictions, aiding developments towards precision medicine.

### Aims, objectives and hypotheses

This study aims to develop an effective, clinically relevant, survival prediction in sCJD using data available at or near the point of diagnosis.

We defined the following hypotheses to achieve this aim:

1. Survival in sCJD can be predicted using data at or near the point of diagnosis. We analysed data collected during the routine diagnostic process of sCJD, including information from the 2017 International CJD Surveillance Network diagnostic criteria.
2. Deep learning methods may improve on traditional survival analysis methods (CoxPH). We compared traditional statistical methods and novel machine-learning methods in parallel.
3. Survival prediction methods can be interpretated for clinical relevance and transparency. Interpretable machine-learning techniques were employed to explain candidate models and their predictions.

## Methods

This study follows reporting guidelines from “Recommendations for Reporting Machine Learning Analyses in Clinical Research” [27] and “REporting of studies Conducted using Observational Routinely-collected health Data (RECORD)” [28].

### Data source

#### Study population

We used clinical surveillance data from the United Kingdom (UK) National CJD Research and Surveillance Unit (NCJDRSU). We included data from consecutive cases of probable or definite sCJD diagnosed according to the 2017 International CJD Surveillance Network diagnostic criteria in a 5 year period between 01/01/2017 and 01/01/2022. The UK NCJDRSU has demonstrated high rates of case ascertainment, linked to detailed clinical phenotyping using specified assessment protocols [29].

As part of national surveillance activity, suspected cases of prion disease were referred from across the UK to the NCJDRSU for specialist assessment. Cases were assessed by a physician, either in-person at the referring hospital, in the community, or remotely using telehealth [30]. Details including history of presentation, past medical history, and routine investigations were curated using a standardised surveillance questionnaire, and stored in the NCJDRSU database.

#### Sample size and censoring

In this context censoring denotes cases which have been lost to follow-up, or cases which were still alive at the time of data extraction. Because sCJD is a rapidly progressive and fatal illness, censoring is usually very low. Censored cases were not included as it is not possible to evaluate very small number of censored cases when conducting cross validation (where the total number of censored cases is less than the number of folds). Bias from this exclusion would likely be negligible.

#### Target variable

The target variable for our model was overall survival time, defined as the number of months elapsed between the onset of symptoms attributed to sCJD, and death.

#### Feature variables

Twenty one features extracted for model input are detailed in Table 1. These include information from the 2017 International CJD Surveillance Network diagnostic criteria, as well as other routinely collected data at or near the point of diagnosis. Chosen features were contemporaneous with the point of diagnosis to reduce potential recall bias. An a priori feature selection process was not conducted as ANNs can incorporate intrinsic feature selection.

**Table 1:** Features for model input, including routinely collected data from the 2017 International CJD Surveillance Network diagnostic criteria for sCJD, and other data available at or near point of diagnosis

Feature	Description
2017 International CJD Surveillance Network diagnostic criteria	
Symptoms at diagnosis	
Myoclonus	Binary
Visual disturbance	Binary
Cerebellar features	Binary
Pyramidal features	Binary
Extrapyramidal features	Binary
Akinetic mutism	Binary
Cognitive impairment	Binary
Psychiatric disturbance	Binary
Behavioural disturbance	Binary
CSF RT-QuIC	Binary (positive or negative)
CSF 14-3-3	Binary (positive or negative)
MRI findings	as assessed by expert neuroradiologist (D.S.)
Impression	Categorical (positive for sCJD, suspicious but not diagnostic, or negative for sCJD)
Basal ganglia signal change	Binary
Thalamic signal change	Binary
Cortical areas affected	Integer
PWCs on EEG	Binary
Other data at or near point of diagnosis	
Age of onset (years)	Float (date of symptom onset – date of birth)
Sex	Binary (male or female)
Polymorphism at codon 129 of the *PRNP* gene	Categorical (MM, MV, or VV)
CSF S100b (ng/ml)	Float
CSF total protein (g/L)	Float

*CSF* cerebrospinal fluid, *RT-QuIC* real-time quaking-induced conversion, *MRI* magnetic resonance imaging, *EEG* electroencephalogram, *PWCs* periodic sharp wave complexes, *MM* methionine homozygosity, *MV* methionine-valine heterozygosity, *VV* valine homozygosity

#### Ethical approval

This study used secondary data from the UK national CJD surveillance, which has been advised by local ethics committee as essential for public health surveillance. Additional informed consent for use of this data for public health research was also obtained.

#### Data availability

The dataset used in this study is available upon request to the NCJDRSU subject to satisfactory data access requirements. This is to protect individual’s privacy due to sCJD’s status as a rare diagnosis.

#### Candidate methods

To compare traditional linear statistical models with a deep-learning implementation, we trained traditional CoxPH models and an ANN model in parallel.

#### Cox proportional hazard models

CoxPH models were trained as linear models implemented in scikit-survival [31]. Two CoxPH models were used; a naïve CoxPH model which incorporated all features, and a CoxPH model with L1 penalty for feature selection (Cox-LASSO).

#### Deep learning method

A neural network implementation of multitask logistic regression for survival analysis (NMTLR) was selected [32]. This approach divides time into a series of time intervals (100 in our model). An ANN is trained on the probabilities of survival at each interval. This approach was chosen because a neural network is flexible and able to learn non-linear interactions between features. The proportional hazards assumption is also bypassed as survival probabilities are modelled in time intervals, removing the need for a baseline hazard function. Time-varying effects of features are also learned naturally as a result. NMTLR has performed favourably in a benchmark of survival analysis in clinical and omics data [22].

### Model training

#### Training and testing sets

**Fig. 1:**
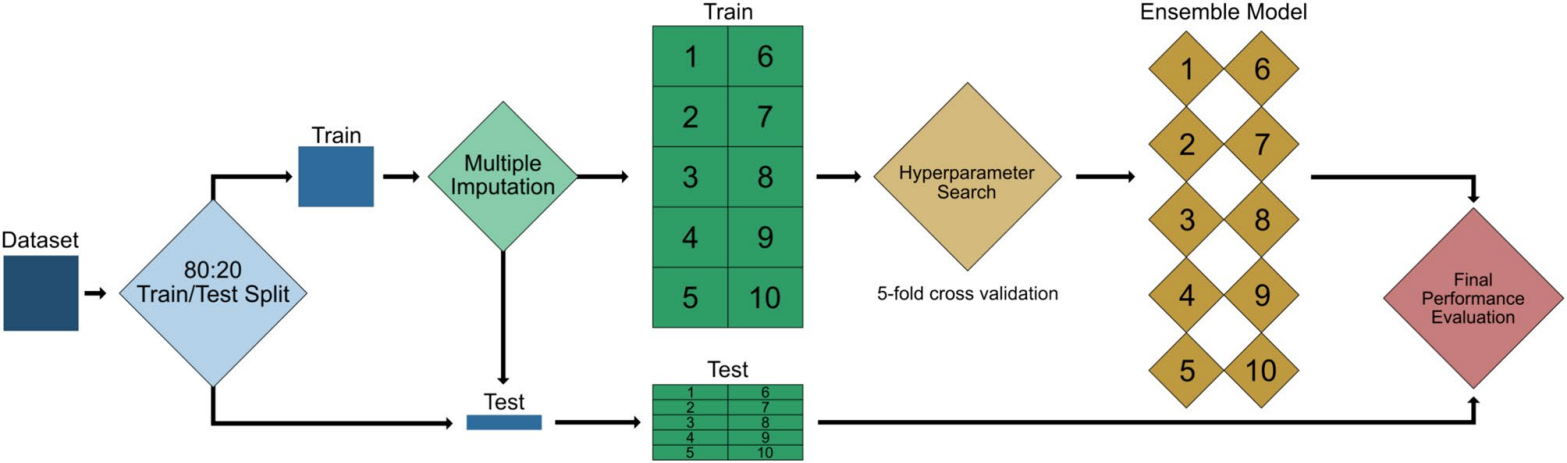
Summary of training and evaluation pipeline. The dataset was divided into an 80% training set and 20% test set. Multiple imputation with chained equations (MICE) was conducted to generate ten imputed datasets. Hyperparameter search was conducted using five-fold cross validation across the ten imputed datasets to independently train an ensemble of ten models. Finaly performance evaluation was conducted using the 20% test set

The dataset was divided in 8:2 ratio in training and testing sets in chronological order, with the earliest cases assigned to the training set, and the latest cases to the testing set (Fig. 1). This was done to demonstrate the generalisability of the model to prospective data and assessment methods.

#### Missing data

Fifteen percent of all entries were missing in our dataset. A multiple imputation with chained equations (MICE) approach was implemented to account for this missingness using miceforest [33]. Existing distribution and relationships in the data are used to generate ten plausible versions of the dataset. Ten independent models are trained and evaluated on each imputed datasets. Predictions and metrics from each independent model are subsequently pooled. The final model is therefore an ensemble of ten independently trained models.

#### Data transformation

Binary and categorical features were one-hot encoded, with one dummy variable dropped from each feature to avoid multi-collinearity. For NMTLR, all features were scaled by standardisation and normalisation to facilitate stable training.

#### Hyperparameter tuning

Details of hyperparameter tuning procedures for NMTLR can be found in Appendix Table 1.

### Model evaluation

For estimation and comparison of performance and generalisability, we calculated commonly reported metrics in survival analysis on the testing set. All metrics were calculated using implementations from scikit-survival [31].

#### Concordance index

Concordance index (CI) is the commonest reported performance metric in survival analysis [34]. CI provides a measure of how well the model ranks individuals according to their survival probabilities. It is therefore a limited performance metric for survival prediction, as it does not provide information on calibration, how well the model predicts the actual value of survival probability.

#### Brier score and integrated Brier scores

Brier score represents the mean squared distances between an observed survival time and predicted survival probability [35]. It can therefore provide information on model calibration as well as concordance. Brier score in survival analysis is time-dependent [BS(t)], which we report using Brier score plot over survival times. To obtain an overall measure of performance across all survival times, the integral of Brier scores from all available timepoints, known as the integrated Brier score (IBS), was also reported. IBS was used as the optimisation objective for Optuna hyperparameter tuning.

#### Cumulative/dynamic area under curve scores

Area under curve (AUC) scores are a measure of an estimator’s sensitivity and specificity. In survival analysis events (death) do not recur in an individual, so AUC is adapted and calculated on cumulative cases (individuals who experience an event at time *t*) and dynamic controls (individuals who experience an event after time *t*) [36]. Therefore AUC is also time-dependent [AUC(t)] in survival analysis. We reported AUC scores at 5 months [AUC(5)] and 10 months [AUC(10)] as overall measures of model performance, alongside plots of AUC(t) to provide measures of model performance over survival times. 5 and 10 months represent particularly important timepoints in sCJD disease course, with 5 months being near the median disease duration, and 10 months being the point at which an individual is often considered to be a “long survivor” in practise.

#### Confidence interval and significance testing

The standard errors of our pooled performance metrics cannot be calculated directly using pooling methods for multiple imputation (known as Rubin’s Rules) [37, 38]. Confidence intervals and hypothesis testing were instead conducted using bootstrapping, with pooling of estimates and standard errors performed using the recommended “MI Boot” method [37]. 1000 iterations of bootstrapping were conducted for each candidate model to generate a bootstrap distribution of performance metrics. These distributions were then used to estimate the standard error, with standard Rubin’s Rules used to construct confidence intervals and conducted pooled two-sided *t* tests on differences in performance between candidate models. Alpha was defined as 0.05.

#### External validation

Consecutive cases of sCJD from a single national cohort were included in the dataset, so external validation would only be possible using other international cohorts. Internal prospective validation and external validation with other national data would therefore be important next steps, which is beyond the scope of this study.

### Model explanation

Candidate models were interpreted using permutation feature importance. Individual predictions were explained using SHapley Additive exPlanations (SHAP) values [39]. Feature interaction analyses were conducted with partial dependence plot (PDP) and individual conditional expectation plots (ICE). Values for calculating partial dependence were predicted risk scores. Implementation from scikit-learn in Python was used [40].

### Clinical examples

Three clinical cases from the testing sets are used as illustration of clinically relevant model usage. Clinical history and input variables are provided.

### Reproducibility

Code developed for this model pipeline can be publicly accessed in its GitLab repository [41].

## Results

**Fig. 2:**
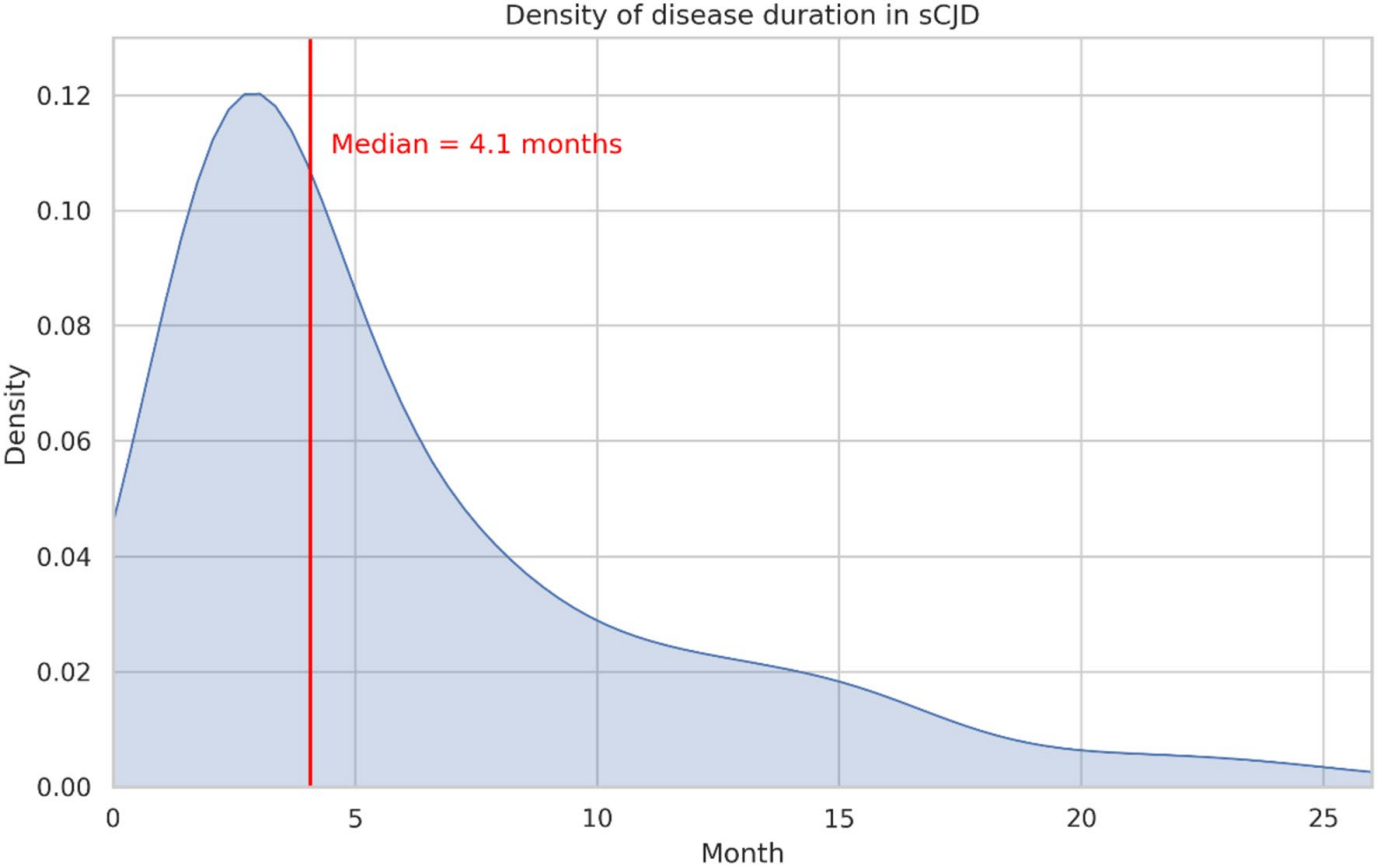
Density of disease duration in the study cohort of 655 individuals with sCJD. Red line marks the median disease duration at 4.1 months

Six hundred and sixty four individuals met inclusion criteria. 1.4% (*n* = 9) individuals were censored and removed from analysis. Therefore, our final analysis cohort consisted of 655 individuals. Median disease duration (symptom onset to death) was 4.1 months (SD = 6.9 months) (Fig. 2), and median diagnostic latency (symptom onset to diagnosis) interval was 1.1 months (SD = 5.1 months). 16.3% (*n* = 107) survived beyond 12 months, and 1.8% (*n* = 12) survived beyond 24 months.

### NMTLR versus Cox models

Statistics and 95% confidence intervals pooled from ensemble of ten models trained on ten imputed datasets using MI Boot method from Schomaker and Heumann 2018.

### Best NMTLR model

The best performing NMTLR model was an ensemble of 3-layer multilayer perceptrons. Selected hyperparameters for the ten individual models are detailed in Appendix Table 1.

### Overall performance

Results of model evaluation are detailed in Table 2. All candidate models demonstrated better performance compared to a random model. This shows that effective survival predictions can be made using routinely collected data at or near the point of diagnosis. NMTLR showed modest performance improvement over both CoxPH models on all metrics, but these differences were not shown to be statistically significant (*p* > 0.05).

**Table 2:** Performance metrics for NMTLR, CoxPH and Cox-LASSO models, with reference values from a random useless model for comparison

Model	C-index ± 95% CI	AUC(5) ± 95% CI	AUC(10) ± 95% CI	IBS ± 95% CI
Random model	0.5000	0.5000	0.5000	0.2500
NMTLR	0.732 ± 0.022	0.866 ± 0.031	0.872 ± 0.032	0.079 ± 0.012
CoxPH	0.730 ± 0.018	0.852 ± 0.030	0.863 ± 0.031	0.083 ± 0.007
Cox-LASSO	0.734 ± 0.016	0.857 ± 0.028	0.859 ± 0.026	0.082 ± 0.006

95% confidence intervals are calculated using the MI Boot method [37]. NMTLR showed modest non-significant performance improvement over both CoxPH models on all metrics (*p* > 0.05)

### Time-dependent performance

**Fig. 3:**
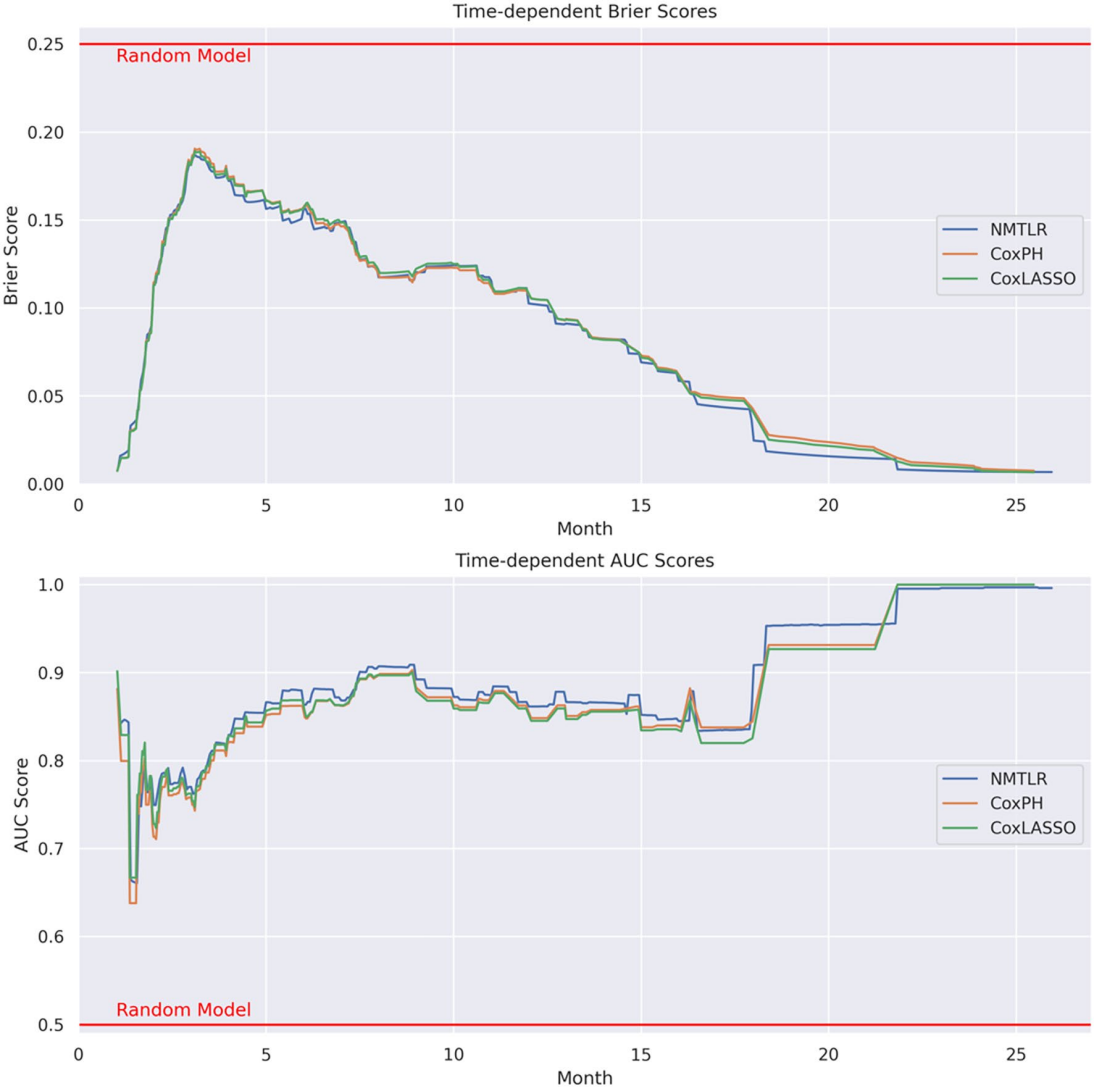
Time-dependent Brier scores and AUC scores for the three candidate models. Performance by Brier score and AUC of all three models drops in the initial months, with gradual improvement over survival time. AUC remains relatively stable between 0.8 and 0.9 until late into survival times when nearly all events have occurred

Figure 3 shows the candidate model’s overall survival times. In the early months of survival times, we can observe a drop in performance in time-dependent Brier score (with a peak close to 0.2) and then gradual improvement over survival times. Time-dependent AUC scores remain relatively stable at around 0.8 to 0.9, until very late into survival times when nearly all events have occurred.

### Feature importance

**Fig. 4:**
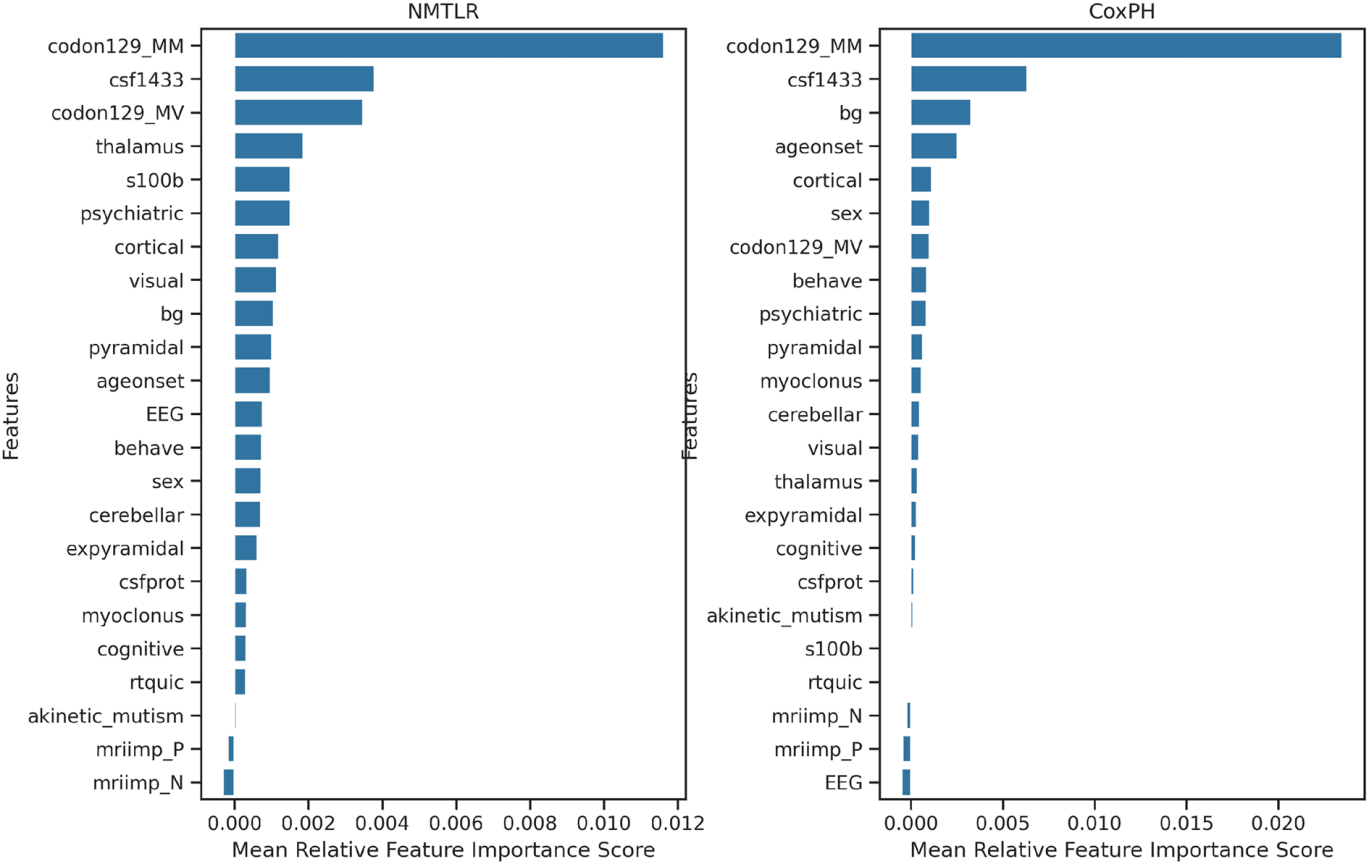
Permutation feature importance in NMTLR (left) and CoxPH (right). Codon 129 polymorphism and CSF 14-3-3 were ranked as the most important predictive features in both models

Figure 4 details permutation feature importance scores in NMTLR and naïve CoxPH models. In both models, polymorphism at codon 129 of the *PRNP* gene was found to the most important feature, followed by CSF 14-3-3. Other relatively less important features also contributed to predictions, including specific MRI features, age of onset, and symptoms present at diagnosis. MRI impression and CSF RT-QuIC were ranked as unimportant by both models.

### Feature interaction

**Fig. 5:**
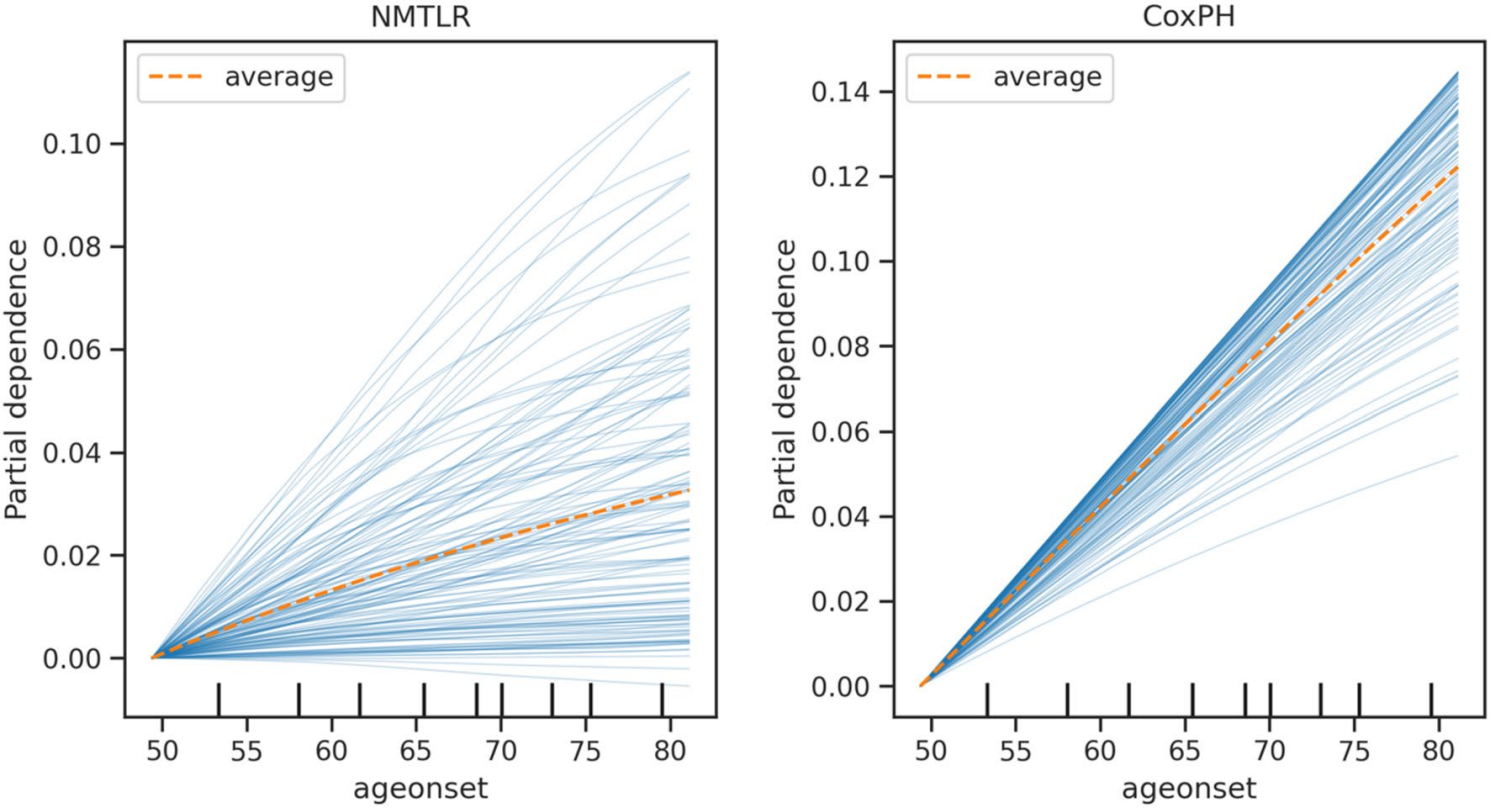
Partial dependence and individual conditional expectation plots of age of onset in NMTLR and CoxPH

To illustrate the nonlinearities captured by NMTLR, Fig. 5 shows the PDP/ICE plots for age of onset in NMTLR and CoxPH. For NMTLR, the relationship between age of onset and its effect on survival is non-linear, whereas the CoxPH model can only represent linear relationships between features and survival.

**Fig. 6:**
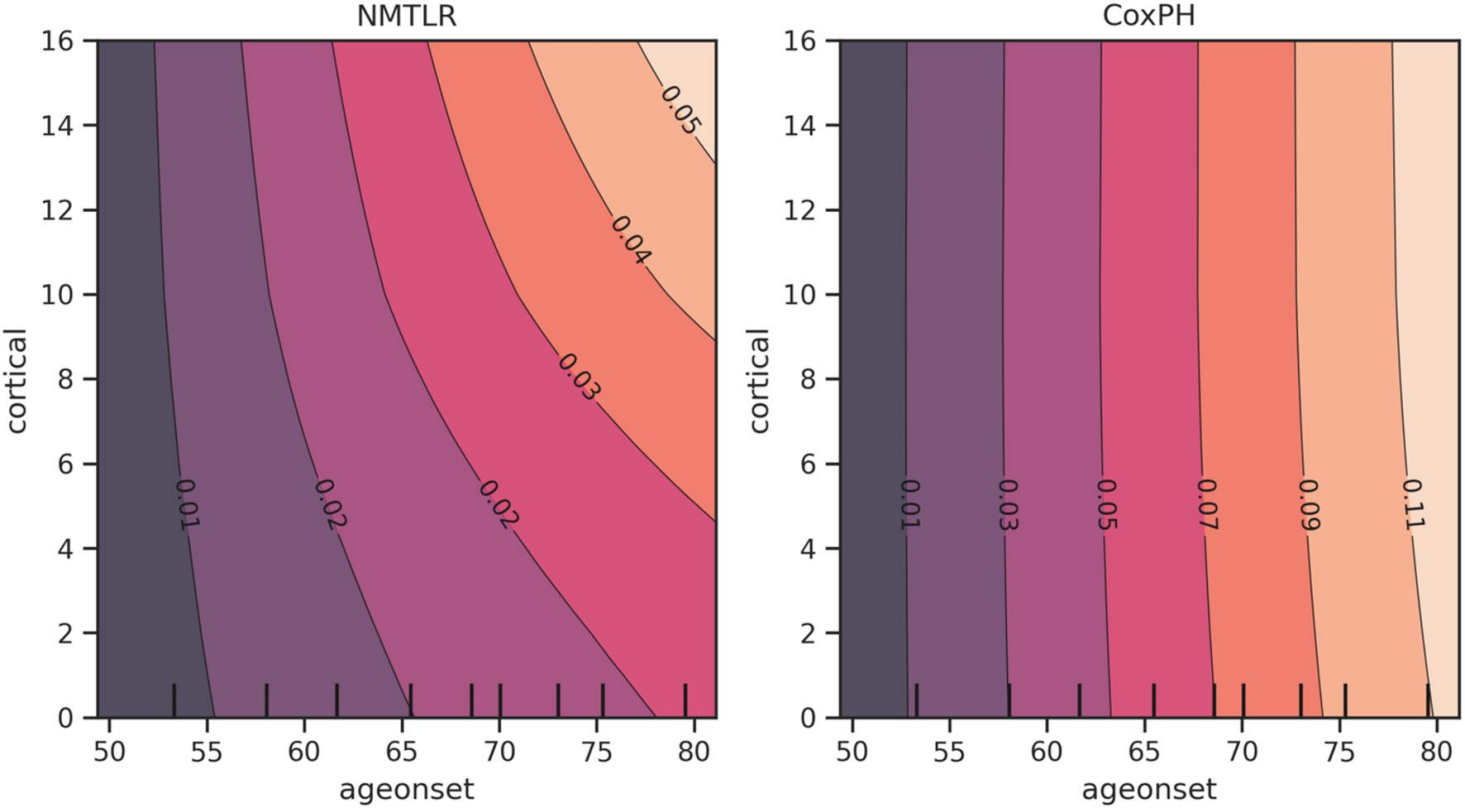
Multivariate partial dependence plots for NMTLR and CoxPH, showing feature interactions between age of onset and the number of cortical areas affected on MRI

The multivariate PDP plots in Fig. 6 illustrates feature interaction between age of onset and the number of cortical areas affected on MRI (cortical). In the CoxPH model, the effect on survival is linear with no feature interactions captured.

### Clinical examples with prediction explanations

**Fig. 7:**
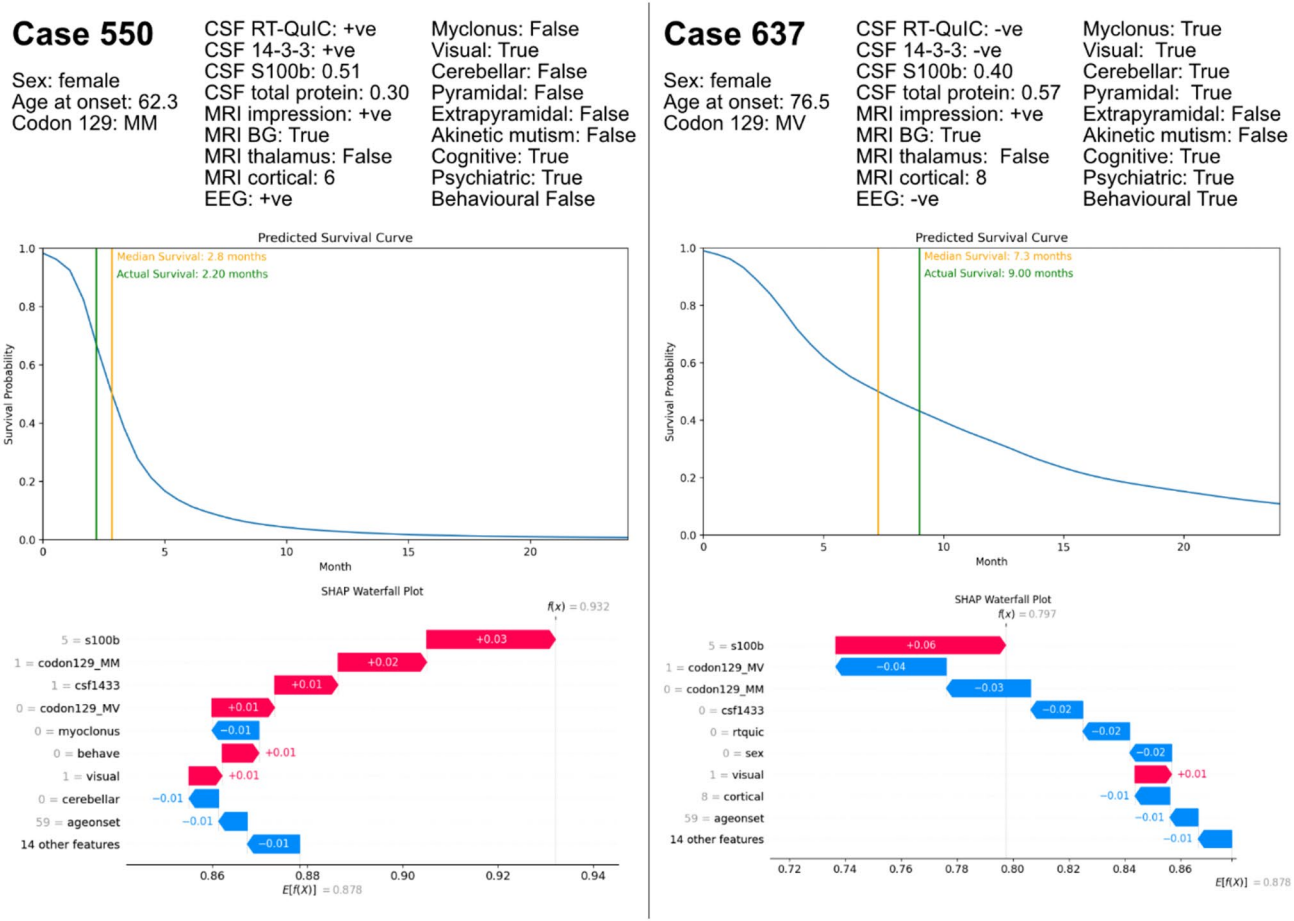
Examples illustrating clinically relevant survival predictions and explanations for case 550 and 637 from the testing set. Green line indicates actual survival, orange line indicates predicted survival. SHAP waterfall plots explain each prediction by summarising features effects on risk scores for an individual case

Figure 7 shows two examples of how this model may be used for clinically relevant survival predictions in sCJD using the NMTLR model. The model outputs a predicted survival function, quantifying survival probabilities at each point in time. Clinically relevant individual prediction explanation is provided in the form of a SHAP waterfall plot, summarising the features that had the greatest effect on risk scores for a particular case. In this example, s100b and codon 129 polymorphism showed the strongest effect on individual survival. These survival predictions are in-keeping with the clinical phenotypes seen in MM and MV sCJD [7].

## Discussion

We present the development of an integrated and multimodal survival prediction model to enhance clinical care of sCJD. Our results demonstrate that effective survival predictions can be made at or near the point of diagnosis using clinical data that are readily available from the routinely collected diagnostic information. Moreover, we establish the use of deep-learning methods in multimodal clinical data of sCJD, demonstrating performance and practical advantages over existing models in literature.

### ANN versus traditional approaches

To the best of our knowledge, this study was the first to investigate an ANN in the study of prion disease. Our results showed that NMTLR produced results with only a modest performance increase over CoxPH models. This was despite model diagnostic results demonstrating NMTLR’s ability to model non-linear interactions and bypass the proportional hazards assumption of CoxPH (Appendix Table 1). This corroborates recent benchmarks of survival models, demonstrating the lasting power and utility of the CoxPH model [22]. Therefore, these modelling methods are all valid to employ in this dataset.

However, ANNs have advantages in the analysis of unstructured data, such as neuroimaging data and medical records. Our model and approach utilised structured tabular data, where CoxPH has excelled for many decades. As healthcare data increase in volume and complexity, deep ANNs will facilitate more powerful models for survival prediction. We argue that our ANN implementation is a promising step and demonstrates the potential for ANN methods in both prion diseases and neurodegenerative conditions.

#### Feature importance

Both NMTLR and CoxPH models identified codon 129 polymorphism as the most significant predictive feature. This is in keeping with findings from existing studies [7, 11, 12]. CSF 14-3-3 was also shown to be significant in both models, which has also been used as a variable in previous prognostic models [19, 20].

In both models, a subset of MRI features were found to be relatively more important predictive variables than age of onset: thalamic signal change in NMTLR and basal ganglia signal change in CoxPH. sCJD subtypes have previously been shown to have characteristic MRI lesion profiles that can facilitate radiological subtype diagnosis [42]. Our work further demonstrates that neuroimaging data can be integrated to provide clinically significant phenotypic information.

MRI impression and CSF RT-QuIC were unimportant features in both models. This is likely because they are categorical variables with significant positive class imbalance (90% positive MRIs and 94% positive RT-QuIC), which limits their discriminatory effect.

### Comparison with existing models

Llorens and colleagues presented a prognostic model for overall survival in sCJD using CoxPH in 2020 [20]. Their model conducted complete case analysis on 1226 cases of sCJD from the German national surveillance programme and reported a CI of 0.686 (95% CI 0.665–0.707) using a tenfold cross-validation approach. Our models demonstrate significant performance increase over their model, perhaps due to a larger number of relevant features, such as neuroimaging and symptomatology data. Furthermore, the inclusion period for Llorens and colleagues’ study ended in 2017 when the international consensus criteria was updated, limiting prospective utility of their model given its bias towards cases ascertained with now outdated diagnostic criteria.

Nihat and colleagues employed a different approach to their prognostic model in 2022, by modelling both survival and escalated care status in individuals diagnosed with sCJD [19]. The study reported AUC scores of 0.94, 0.92 and 0.91 for survival at 10, 30 and 100 days from diagnosis. Key differences in our approach provides practical and statistical advantages over Nihat and colleague’s model. In their study, time-to-event was defined from the point of diagnosis instead of overall survival. This approach neglects symptomatic disease which may have elapsed prior to diagnosis. Whilst acknowledging factors such as recall bias is date of symptom onset, this information is important to incorporate given the previously reported diagnostic delay associated with sCJD [43]. In addition, the National Prion Monitoring Cohort used in Nihat and colleagues’ study is biased to individuals with longer survival, due to their exclusion of individuals with higher levels of neurodisability at the point of diagnosis (MRC Scale < 5) from 2015 [44]. This limits generalisability and utility of their model in most individuals with sCJD, who progress rapidly and often present to specialist services in advanced stages. Finally, the reporting of performance metrics was limited, with no report of other commonly used performance metrics in survival analysis, and no reporting of performance metrics beyond 100 days. This limits the utility of their model given that the median survival in sCJD is 120–150 days [1].

### Strengths and limitations

Our models utilise data from consecutive sCJD cases from a national surveillance programme, increasing the generalisability of our models. Missingness of the dataset was low at fifteen percent of entries, with multiple imputation allowing for incorporation of all data from the dataset and estimation of the uncertainty of missingness. Evaluation was conducted using a comparable testing set of prospective cases, demonstrating performance to new incoming data. Our model outputs are clinically relevant, with prediction-level explanations that facilitate model transparency and interpretation.

The dataset we utilised was based in the UK, with a large proportion of individuals of white British descent, which limits the international generalisability of these models [18]. Incorporation of data from international collaborators would facilitate external validation, and potentially improve performance and relevance to other ethnicities. The clinical features of our models, although based on international consensus diagnostic criteria, are subject to biases from different clinician raters. We mitigated this using standardised assessment protocols and questionnaires in data collection. Further improvements and validation of these assessment procedures would ensure limited bias in subsequent analyses.

Cross-sectionally collected clinical and epidemiological data were used for this study to illustrate the utility of models to predict survival at the point of diagnosis. Features contemporaneous with the time of diagnosis were chosen, but some bias is still likely to be present as data was collected retrospectively. In addition, a model that incorporates additional prospective longitudinal information using deep-learning methods such as reinforcement learning may further increase performance, especially in known “long survivors”.

### Future work

We aim to validate our model further by deploying this model in routine national prion disease assessments and conduct internal prospective validation. External validation and remodelling can also be conducted in collaboration with other international CJD surveillance programmes. An internationally validated survival prediction model for sCJD would be of immense value to clinical management and research. Incorporation of additional data in our deep-learning approach may further increase performance. Disease severity measures (such as the MRC Prion Disease Rating Scale [44]) and staging were not routinely conducted but may provide additional information that would benefit predictions. Codon 129 polymorphism in the *PRNP* gene was by far the most important feature in our models. MRI-related features, such as the presence of thalamic signal change, also appeared to exert significant influences on survival in NMTLR. CSF tau has been shown to be an important determinant of survival not only in prion disease, but also other neurodegenerative disorders such as Alzheimer’s disease [45, 46]. Whilst these biomarkers are presently not routinely incorporated in international consensus diagnostic criteria for sCJD, analysis and inclusion of other biomarkers, genomics and neuroimaging data may further boost performance of multimodal survival prediction models in the future.

This study focussed on modelling overall survival.

## Conclusion

Effective, interpretable and clinically relevant survival predictions in sCJD can be made at or near the point of diagnosis. Novel ANNs such as NMTLR hold potential for including more voluminous and complex data to improve predictions and facilitate personalised care. Further clinical and collaborative validation work would contribute to the development of increasingly accurate survival prediction models. Our study highlights the potential for these approaches in both prion diseases and neurodegenerative conditions. Clinical adoption will lead to improved prognostication, informing clinical and end-of-life interventions, care planning, and stratifications to future clinical trials in prion diseases.

## Appendix

See Table 1.

**Table 1:** NMTLR hyperparameter tuning

	Models									
Hyperparameter	0	1	2	3	4	5	6	7	8	9
Learning rate	0.018	0.018	0.044	0.029	0.033	0.043	0.020	0.019	0.044	0.072
Epochs	144	169	176	153	150	88	135	131	165	162
Weight decay	0.110	0.070	0.020	0.080	0.080	0.040	0.090	0.080	0.100	0.040
Hidden layer 1 nodes	121	48	375	375	206	251	276	271	432	57
Hidden layer 2 nodes	223	237	95	472	281	461	180	168	480	211
Dropout	0.720	0.670	0.460	0.760	0.810	0.800	0.730	0.700	0.730	0.700

Ten models were tuned independently to the corresponding multiply imputed training sets using the Optuna package in Python [47]. A five-fold cross validation procedure was utilised within the training set to utilise all available training data, and reduce bias in validation metrics. Each model underwent 500 iterations of optimisation to discover ten best models to form the final ensemble of ten models. The hyperparameters used in the final ensemble are detailed below.
